# Redox reactions in chronic pain: mechanisms and relevance in fibromyalgia

**DOI:** 10.3389/fpain.2025.1593908

**Published:** 2025-05-13

**Authors:** Tim Ho, Mark Ryan, Jonas Holle

**Affiliations:** ^1^ Sydney Meical School, Faculty of Medicine and Health, University of Sydney, Sydney, NSW, Australia; ^2^ Cingulum Health, Sydney, NSW, Australia

**Keywords:** fibromyalgia, oxidative stress, mitochondrial dysfunction, redox imbalance, neuroinflammation

## Abstract

Fibromyalgia (FM) is increasingly recognized as a disorder driven by oxidative stress, mitochondrial dysfunction, and neuroinflammation, contributing to pain sensitization and fatigue. This review explores the role of redox imbalance in FM and evaluates potential therapeutic interventions. A scoping literature search was conducted using PubMed, Scopus, and Google Scholar. Findings indicate elevated oxidative stress markers (MDA, 4-HNE), impaired antioxidant defenses [CoQ10 (Coenzyme Q10), SOD, catalase], and mitochondrial dysfunction in FM patients. Preclinical and small-scale clinical studies suggest potential benefits of NRF2 activation, high-dose thiamine, CoQ10, molecular hydrogen, and oxygen-ozone (O_2_O_3_) therapy. However, human trial evidence is limited, and standardized treatment protocols are lacking. Given the absence of robust RCTs, oxidative stress modulation in FM remains investigational. Future research should prioritize high-quality RCTs to establish the efficacy, safety, and clinical application of redox-targeted therapies.

## Introduction

Fibromyalgia (FM) is a chronic pain syndrome characterized by widespread musculoskeletal pain, profound fatigue, and cognitive dysfunction. While its precise etiology remains unclear, growing evidence implicates oxidative stress and mitochondrial dysfunction as key contributors to its pathophysiology (1, 2).

Oxidative stress results from an imbalance between reactive oxygen species (ROS) production and antioxidant defenses, leading to cellular damage, lipid peroxidation, mitochondrial dysfunction, and neuroinflammation—all of which may exacerbate pain sensitization and fatigue in FM (3, 4).

Mitochondrial dysfunction in Redox imbalance is marked by decreased CoQ10 levels, reduced mitochondrial DNA content, and impaired electron transport chain activity, leading to excessive ROS production and decreased ATP synthesis. These bioenergetic deficits contribute to muscle and neuronal fatigue (1, 2).

This scoping review aims to examine the role of oxidative stress and mitochondrial dysfunction in FM, with a focus on redox imbalance, neuroinflammation, and potential therapeutic interventions targeting these pathways. This review also seeks to identify gaps in knowledge and highlight potential directions for future research into redox-targeted treatments for FM.

## Method

This scoping review followed the PRISMA-ScR guidelines. A systematic search was conducted across Medline (Ovid), Embase, and Web of Science from inception to January 2025 to identify relevant literature on oxidative stress, mitochondrial dysfunction, and antioxidant therapies in fibromyalgia (FM).

Boolean operators and Medical Subject Headings (MeSH) were applied to refine the search strategy. For example, the Medline search included:

exp Fibromyalgia/or fibromyalgia.mp.

AND

exp Oxidative Stress/or oxidative stress.mp. or redox imbalance.mp.

Similar expressions were adapted for Embase and Web of Science. “OR” was used to combine synonyms or related terms within a concept, and “AND” was used to combine major concepts across themes. The full search strategy for each database is provided in the Supplementary Material.

This review was not registered on PROSPERO as it is a scoping review. However, the protocol was developed *a priori* and followed a standardized framework.

### Study selection was guided by the following PICO-derived questions

In individuals with fibromyalgia, does increased oxidative stress or mitochondrial dysfunction (compared to healthy controls) correlate with greater symptom severity and inflammatory biomarkers?

In fibromyalgia, do alterations in phospholipid metabolism (e.g., reduced lysophosphatidylcholines, increased lysophosphatidylethanolamines) contribute to neuroinflammatory signaling and pain sensitization?

Do individuals with fibromyalgia exhibit reduced antioxidant capacity—such as lower superoxide dismutase (SOD), catalase, or CoQ10—that correlates with increased oxidative stress and symptom severity?

Do redox-modulating therapies (e.g., thiamine, CoQ10, NRF2 activators, molecular hydrogen), when compared to placebo or standard care, improve pain, fatigue, sleep, or oxidative stress markers in FM?

### Eligibility criteria

#### Studies were included if they met the following criteria

##### Population

Human studies in individuals with fibromyalgia or preclinical models relevant to oxidative stress, mitochondrial dysfunction, or redox-modulating interventions.

##### Study design

RCTs, systematic reviews, meta-analyses, observational studies, and mechanistic preclinical studies.

##### Exclusions

Case reports with fewer than 10 patients, non-English publications, and studies not specific to fibromyalgia.

### Study selection and data extraction

A total of 446 articles were retrieved. Two reviewers independently screened titles and abstracts for inclusion, with conflicts resolved by a third reviewer. Of these, 169 articles were included after full-text review.

#### Data were extracted into a standardized table capturing

Study design, Sample size and population, Intervention or exposure (e.g., antioxidant agent, mitochondrial marker), Outcomes (e.g., pain, fatigue, oxidative markers), Key findings relevant to the PICO questions above.

### Quality assessment

Randomized controlled trials were assessed using GRADE methodology. Observational and preclinical studies were retained for mechanistic insights but assigned lower levels of certainty in clinical translation.

Chronic pain involves persistent nociceptor and microglial overactivation, which leads to electron transport chain (ETC) dysfunction in neuronal mitochondria and excessive production of reactive oxygen species (ROS) and reactive nitrogen species (RNS). This disruption initiates a vicious cycle of oxidative stress, mitochondrial dysfunction, and neuroinflammation, further sensitizing pain pathways. In overactivated nociceptors, mitochondrial ETC dysfunction results in electron leakage, leading to the formation of superoxide (O₂^−^·). This superoxide is rapidly converted into hydrogen peroxide (H₂O₂), which, in the presence of Fe^2+^, generates highly reactive hydroxyl radicals (·OH) through the Fenton reaction. Similarly, activated microglia contribute to oxidative stress by upregulating NADPH oxidase 2 (NOX2), which generates O₂^−^·, and inducible nitric oxide synthase (iNOS), which produces nitric oxide (NO). The reaction between NO and O₂^−^· forms peroxynitrite (ONOO^−^), a potent RNS capable of nitrating and damaging cellular structures. The accumulation of ROS and RNS results in lipid peroxidation, where polyunsaturated fatty acids (PUFAs) in neuronal membranes undergo oxidative damage, compromising cell integrity and function. Additionally, these reactive species induce DNA oxidation, leading to mitochondrial and nuclear DNA mutations, further impairing ATP production and exacerbating ETC dysfunction. As a result, mitochondria become increasingly inefficient, producing more ROS and RNS, perpetuating neuronal injury, microglial activation, and chronic inflammation. Although antioxidant defense mechanisms [glutathione peroxidase (GPx), peroxiredoxin (Prx), and catalase (Cat)] work to neutralize ROS, their capacity is often overwhelmed in chronic pain states. This imbalance sustains a self-amplifying loop of oxidative stress, neuroinflammation, and mitochondrial dysfunction, reinforcing chronic pain pathophysiology.

### Oxidative stress, mitochondrial dysfunction, and fibromyalgia

**Figure 1:**
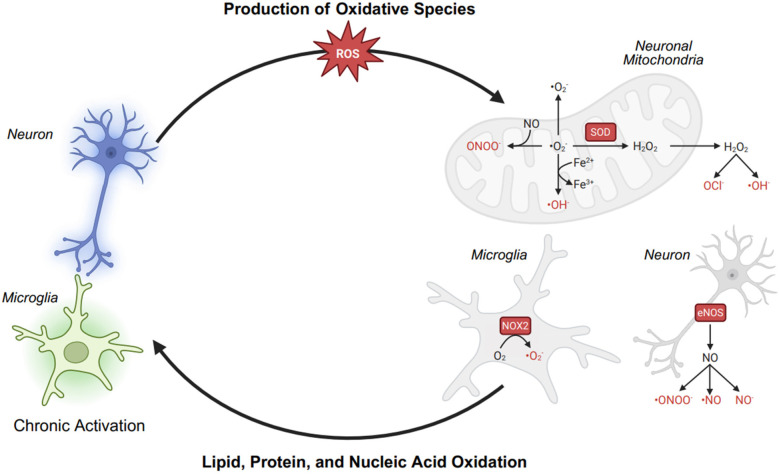
Pathophysiology of overactivation-induced oxidative stress in chronic pain.

Fibromyalgia (FM) is associated with oxidative stress and redox imbalance, contributing to mitochondrial dysfunction, neuroinflammation, and central sensitization (1) (Figure 1).

Elevated lipid peroxidation markers, such as malondialdehyde (MDA) and 4-hydroxynonenal (4-HNE), correlate with symptom severity in observational human studies, suggesting that reactive oxygen species (ROS) drive oxidative damage in FM. ROS, including superoxide (O₂^−^), hydroxyl radicals (OH^−^), and hydrogen peroxide (H₂O₂), are highly reactive molecules that damage lipids, proteins, and DNA. Lipid peroxidation occurs when ROS attack polyunsaturated fatty acids in cell membranes, forming MDA and 4-HNE as toxic byproducts. These reactive aldehydes further disrupt mitochondrial function, activate pro-inflammatory pathways (e.g., NF-κB), and sensitize nociceptive neurons, contributing to neuroinflammation, neuronal dysfunction in FM (1, 5).

Mitochondrial dysfunction in FM is evident from reduced electron transport chain (ETC) activity, lower oxygen consumption in muscle biopsies, and diminished bioenergetic reserve (6). Muscle and neural tissue show decreased PGC-1α and mitofusin-2 (Mfn2) expression, critical regulators of mitochondrial biogenesis and fusion, contributing to ATP deficits and ROS accumulation (7, 8). These changes impair nociceptive threshold regulation and neuronal resilience.

Neuronal and microglial mitochondrial dysfunction in FM impairs electron transport chain (ETC) activity, leading to electron leakage and superoxide (O₂^−^) formation, which contribute to ATP deficits. The mitochondrial impairment in FM is evident from reduced bioenergetic health index (BHI) and increased mitochondrial miRNAs (mitomiR-145-5p) in peripheral blood mononuclear cells (PBMCs), which regulate oxidative stress responses (4). CoQ10 depletion further disrupts electron transfer, and impairing ATP synthesis. In neurons, ATP deficits compromise ion channel function and neurotransmitter release, weakening descending pain inhibition and promoting central sensitization. In microglia, mitochondrial dysfunction shifts them into a pro-inflammatory state, which amplifies neuroinflammation and nociceptive sensitization. An animal study in a reserpine-induced fibromyalgia model demonstrated that TRPA1 activation in glial cells promotes oxidative stress via NADPH oxidase (NOX1), leading to neuroinflammation and nociceptor sensitization (9). In human observational FM studies, increased serum tumor necrosis factor-alpha (TNF-α) levels correlate negatively with CoQ10 and positively with ROS (10). The resultant microglial activation in the central nervous system also reinforces neuroinflammation, contributing to persistent pain and fatigue in FM (2, 10).

Observational studies indeed suggest that oxidative stress disrupts neurotransmitter regulation, particularly serotonin (11). Oxidative stress impairs serotonin signaling by diverting tryptophan to the kynurenine pathway, damaging synthesis enzymes like TPH (tryptophan hydroxylase), and disrupting ATP-dependent neurotransmitter release. Serotonin depletion may increase pain sensitivity, depression, and anxiety. Additionally, deficits in endogenous pain inhibition, particularly within the descending noradrenergic pathway, are implicated in central sensitization. Oxidative stress may impair this pathway, reducing its ability to suppress nociceptive transmission, thereby contributing to exaggerated pain responses in FM (2).

### Lipid metabolism alterations and fibromyalgia

Recent metabolomic studies have highlighted significant alterations in lipid metabolism in FM patients, particularly shifts in phospholipid composition (12). Normally, lysophosphatidylcholines (LPCs) and phosphatidylcholines (PCs) maintain membrane fluidity, facilitate neurotransmitter release, and regulate immune homeostasis. In FM, LPCs and PCs are reduced, while lysophosphatidylethanolamines (LPEs) and triglycerides are elevated, reflecting membrane instability and metabolic dysfunction. These lipid shifts alter microglial signaling, as LPCs normally suppress pro-inflammatory pathways, whereas LPE accumulation triggers Toll-like receptor (TLR) activation, amplifying NF-κB-mediated release of TNF-α, IL-6, and IL-1β. This pro-inflammatory state disrupts neuroimmune balance, increases nociceptor sensitization.

Notably, LPC (16:0), a lipid oxidation product, is elevated in FM and directly activates acid-sensing ion channel 3 (ASIC3) on nociceptors, leading to hyperalgesia in animal models (13). Inhibiting LPC synthesis or blocking ASIC3 reduces pain behavior, suggesting that oxidized lipid species directly contribute to chronic pain signaling in FM (13). These findings suggest that metabolic dysfunction exacerbates oxidative stress and mitochondrial impairment, reinforcing a pathological cycle of inflammation and neuronal hyperexcitability in FM (14).

### Antioxidant defense impairment in fibromyalgia

The imbalance between ROS production and antioxidant defenses is a feature of FM pathophysiology (15). Studies have shown that FM patients exhibit significantly lower levels of total antioxidant capacity and reduced activity of key antioxidant enzymes, including superoxide dismutase (SOD) and catalase. Serum antioxidant levels, particularly CoQ10 and vitamin C, are also decreased in FM patients, further impairing the body's ability to counteract oxidative damage (16). These deficits in antioxidant capacity contribute to increased oxidative stress, leading to mitochondrial dysfunction, inflammation, and heightened pain sensitivity (16, 17).

Several studies have shown that antioxidant enzyme deficiencies, including low SOD, glutathione peroxidase, and catalase, correlate inversely with disease severity measures such as the Fibromyalgia Impact Questionnaire (FIQR), pain scores, and anxiety levels (18, 19). Thiol-disulfide imbalance has also been identified, with decreased native thiol and increased disulfide levels in FM patients, indicating systemic redox stress independent of age or BMI (20).

Further, the nuclear factor erythroid 2-related factor 2 (NRF2) pathway is a master regulator of antioxidant and cytoprotective gene expression, crucial for cellular defense against oxidative stress. Under normal conditions, NRF2 is bound to Kelch-like ECH-associated protein 1 (KEAP1), which facilitates its degradation. In response to oxidative stress, NRF2 dissociates from KEAP1, translocates to the nucleus, and activates genes encoding antioxidant enzymes (e.g., superoxide dismutase SOD) and glutathione synthesis enzymes, reducing reactive oxygen species (ROS) and mitigating oxidative damage (21). In fibromyalgia (FM), NRF2 activity appears impaired, contributing to oxidative damage and neuroinflammation. Preclinical models suggest that NRF2 activation enhances antioxidant enzyme expression, but human clinical evidence in FM is lacking (22, 23).

### Therapeutic interventions targeting redox imbalance in fibromyalgia

Currently, there is a lack of randomized controlled trials (RCTs) directly evaluating redox-modulating interventions targeting the central nervous system (CNS) in fibromyalgia. Growing evidence implicates oxidative stress and mitochondrial dysfunction in fibromyalgia (FM), prompting interest in redox-modulating therapies (24).

High-dose thiamine (600–1,800 mg/day) has shown promise in improving fatigue and pain in FM, likely by enhancing mitochondrial function and reducing oxidative stress (25). Case studies report symptom relief, but robust randomized controlled trials (RCTs) are lacking. Thiamine, essential for ATP production and neurotransmitter synthesis (GABA, acetylcholine), plays a key role. Thiamine deficiency has been linked to hyperarousal and non-restorative sleep, suggesting that addressing oxidative stress and thiamine deficiency may be a therapeutic target for FM-related sleep dysfunction (26, 40). Other antioxidants, including N-acetylcysteine (NAC), resveratrol, and curcumin, have demonstrated anti-inflammatory and neuroprotective effects, further supporting their potential role in FM (27–29).

NRF2-activating agents may improve both pain and sleep in FM by enhancing antioxidant defenses, mitochondrial function, and reducing neuroinflammation (3). An intermittent cold stress (ICS) fibromyalgia mouse model showed that 4-amino-3-(phenylselenyl)benzenesulfonamide (4-APSB), a selenium-sulfa compound with antioxidant properties reduces oxidative stress and neuroinflammation by activating NRF2, leading to enhanced antioxidant defense, lower IL-1β/TNF-α, and reduced pain and depressive-like behaviors (13). NRF2 activation plays a key role in cellular repair during sleep by upregulating antioxidant enzymes (SOD, catalase, HO-1), reducing oxidative damage, and promoting mitochondrial biogenesis, which are critical for neuronal recovery and energy homeostasis. Sleep disruption in FM may impair NRF2-driven antioxidant responses, leading to increased oxidative stress and neuroinflammation, which further exacerbate pain and fatigue (30). Since cognitive behavioral therapy for insomnia (CBTi) can enhance sleep quality and support endogenous antioxidant defenses, combining natural sleep interventions with pharmacological NRF2 activators such as dimethyl fumarate and sulforaphane may reinforce these pathways, stabilizing sleep patterns, reducing pain hypersensitivity, and improving fatigue. Further studies are needed to explore the bidirectional relationship between sleep quality, NRF2 activation, and symptom improvement in FM.

Melatonin supplementation (3 mg/day) has been shown to improve objective and subjective sleep quality, increase 6-sulfatoxymelatonin levels, and enhance total antioxidant capacity in FM patients, suggesting dual chronobiotic and antioxidant benefits (31).

Mitochondrial-targeted therapies such as CoQ10, alpha-lipoic acid (ALA), and carnitine have been investigated for their role in improving ATP production, reducing oxidative stress, and enhancing cellular metabolism. Preliminary clinical studies suggest CoQ10 (300–400 mg/day) improves fatigue and sleep disturbances, but larger trials are needed for confirmation. CoQ10 supplementation, when added to pregabalin, significantly reduced pain and mitochondrial oxidative stress in FM patients, with improved anxiety and brain activity profiles in a small RCT (32).

Oxygen-ozone (O₂O₃) therapy, an intervention that activates NRF2, increases SOD, catalase, and HO-1, while suppressing pro-inflammatory cytokines (IL-6, TNF-α, IL-1β) (16, 33). Small clinical studies suggest it may provide pain relief and functional improvements, but the lack of standardized protocols and high-quality RCTs limits clinical adoption (34). An animal study in a fibromyalgia mouse model demonstrated that Mo₂C nanozyme, a catalytic antioxidant mimicking SOD, catalase, and GPx, restores oxidative balance by scavenging ROS (O₂^−^, H₂O₂), protecting mitochondria, and enhancing ATP production (7, 35). An animal study in a reserpine-induced fibromyalgia rat model found that nano-pregabalin (N-PG) alleviated FM symptoms by enhancing CNS penetration, restoring neurotransmitter balance (Glut, NE, CGRP), increasing antioxidant enzyme activity (e.g., SOD), inhibiting pro-inflammatory transcription factors (e.g., NF-κB), and reducing apoptosis by decreasing caspase-3 (Casp-3) activation (8, 36).

Molecular hydrogen (H₂) is emerging as a potential therapy targeting oxidative stress, mitochondrial dysfunction, and inflammation. As a selective antioxidant, H₂ neutralizes hydroxyl radicals, restoring redox balance—a key mechanism implicated in FM (37). Preclinical studies suggest hydrogen-rich water (HRW), typically administered at 0.8–1.6 ppm H₂ in drinking water (∼5–10 ml/day per mouse), reduces neuropathic pain symptoms, allodynia, and hyperalgesia by modulating oxidative stress and inflammation. HRW upregulates antioxidant enzymes (HO-1, SOD-1) while suppressing pro-inflammatory cytokines (IL-1β, IL-6, TNF-α) and NF-κB activation (38). It also enhances mitochondrial function and ATP-sensitive potassium (K ATP) channel activity, contributing to analgesic and neuroprotective effects. Preclinical models suggest anxiolytic and antidepressant properties, which may be beneficial given the high prevalence of mood disorders in FM. However, clinical trials of molecular hydrogen in fibromyalgia remain lacking, and well-controlled clinical studies are needed to confirm efficacy, optimal dosing, and safety (39).

Future research should prioritize high-quality RCTs to determine the clinical applicability, optimal dosing, and long-term efficacy of these redox-modulating interventions in FM. Key redox mechanisms and associated therapeutic interventions are summarized in Table 1.

**Table 1:** Key redox mechanisms contributing to fibromyalgia.

Mechanism	Key features	Impact on fibromyalgia	Therapeutic interventions
Lipid peroxidation	Elevated MDA and 4-HNE levels correlate with symptom severity, leading to oxidative damage to cell membranes and proteins.	Increases neuroinflammation and neuronal dysfunction, amplifying pain perception.	N-acetylcysteine (NAC), resveratrol, curcumin, molecular hydrogen
Mitochondrial dysfunction	Excess ROS, impaired electron transport, and CoQ10 depletion contribute to energy deficits and pain sensitization.	Leads to ATP depletion, fatigue, and increased central sensitization.	CoQ10, alpha-lipoic acid, carnitine, Molecular hydrogen.
Neuroinflammation	Microglial activation and increased TNF-α and IL-6 levels sustain chronic neuroinflammation, worsening pain and fatigue.	Sustains chronic pain states and contributes to systemic fatigue.	Molecular hydrogen, oxygen-ozone therapy
Neurotransmitter dysregulation	Serotonin depletion impairs pain modulation and mood stabilization, increasing pain sensitivity, depression, and anxiety.	Enhances central sensitization and psychiatric comorbidities.	Thiamine
Antioxidant defense impairment	Reduced antioxidant enzyme activity (SOD, catalase) and lower CoQ10 and vitamin C levels contribute to oxidative stress.	Promotes mitochondrial dysfunction, inflammation, and heightened pain sensitivity.	Oxygen-ozone therapy, CoQ10, vitamin C, alpha-lipoic acid
NRF2 dysregulation	NRF2 activation is impaired, reducing antioxidant enzyme expression and leading to increased oxidative damage and inflammation.	Weakens cellular antioxidant defenses, worsening oxidative stress and neuroinflammation.	Dimethyl fumarate, sulforaphane, molecular hydrogen (activating NRF2 pathway).

## Conclusion

Fibromyalgia is increasingly recognized as a disorder driven by oxidative stress, mitochondrial dysfunction, and neuroinflammation, contributing to pain amplification and fatigue. While preclinical and observational studies suggest potential benefits of redox-modulating therapies, current clinical evidence remains insufficient for routine implementation. The lack of large, well-controlled RCTs limits the clinical application of oxidative stress modulation in FM. Future research should prioritize high-quality trials to evaluate mitochondrial-targeted interventions, validate redox-based biomarkers, and optimize treatment protocols for improved patient outcomes.
